# Generalized Approach to Translating Exercise Tests and Prescribing Exercise

**DOI:** 10.3390/jfmk5030063

**Published:** 2020-08-12

**Authors:** Carl Foster, James D. Anholm, Daniel Bok, Daniel Boullosa, Giancarlo Condello, Cristina Cortis, Andrea Fusco, Salvador J. Jaime, Jos J. de Koning, Alejandro Lucia, John P. Porcari, Kim Radtke, Jose A. Rodriguez-Marroyo

**Affiliations:** 1 Department of Exercise and Sport Science, University of Wisconsin-La Crosse, La Crosse, WI 54601, USA; sjaime@uwlax.edu (S.J.J.); j.j.de.koning@vu.nl (J.J.d.K.); jporcari@uwlax.edu (J.P.P.); kradtke@uwlax.edu (K.R.); 2 VA Medical Health Care System, Loma Linda, CA 92697, USA; James.Anholm@va.gov; 3 Faculty of Kinesiology, University of Zagreb, 10000 Zagreb, Croatia; daniel.bok@kif.unizg.hr; 4 INISA, Federal University of Mato Grosso do Sul, Campo Grande 79070-900, Brazil; daniel.boullosa@gmail.com; 5 College of Healthcare Sciences, James Cook University, Townsville 4811, Australia; 6 Graduate Institute of Sports Training, Institute of Sports Sciences, University of Taipei, Taipei 111, Taiwan; giancarlo.condello@gmail.com; 7 Department of Human Sciences, Society and Health, University of Cassino and Lazio Meridionale, 03043 Cassino, Italy; c.cortis@unicas.it (C.C.); andrea.fusco@unicas.it (A.F.); 8 Department of Human Movement Science, Movement Sciences Amsterdam, Vrije Universiteit, 1081BT Amsterdam, The Netherlands; 9 Faculty of Sport Sciences, Universidad Europea de Madrid, 28670 Villaviciosa de Odón, Spain; alejandro.lucia@universidadeuropea.es; 10 Research Institute ‘imas12’, Hospital 12 de Octubre, 28041 Madrid, Spain; 11 Department of Physical Education and Sports, University of León, 24071 León, Spain; j.marroyo@unileon.es

**Keywords:** exercise prescription, target heart rate, RPE, Talk Test

## Abstract

Although there is evidence supporting the benefit of regular exercise, and recommendations about exercise and physical activity, the process of individually prescribing exercise following exercise testing is more difficult. Guidelines like % heart rate (HR) reserve (HRR) require an anchoring maximal test and do not always provide a homogenous training experience. When prescribing HR on the basis of % HRR, rating of perceived exertion or Talk Test, cardiovascular/perceptual drift during sustained exercise makes prescription of the actual workload difficult. To overcome this issue, we have demonstrated a strategy for “translating” exercise test responses to steady state exercise training on the basis of % HRR or the Talk Test that appeared adequate for individuals ranging from cardiac patients to athletes. However, these methods depended on the nature of the exercise test details. In this viewpoint, we combine these data with workload expressed as Metabolic Equivalent Task (METs). We demonstrate that there is a regular stepdown between the METs during training to achieve the same degree of homeostatic disturbance during testing. The relationship was linear, was highly-correlated (r = 0.89), and averaged 71.8% (Training METs/Test METs). We conclude that it appears possible to generate a generalized approach to correctly translate exercise test responses to exercise training.

## 1. Introduction

Exercise is a very positive health behavior. As far back as Hippocrates in the 4th century Before Common Era (BCE) and Galen in the 3rd century BCE, the concept of *mens sana in corpore sano* “a healthy mind in a healthy body” has been one of the cornerstones of medical practice. Exercise is particularly beneficial considering that in most of the developed world, heart disease is the leading attributable cause of death (45–50% of deaths), and cancer the 2nd (30–35% of deaths), although their incidence is meaningfully reduced in people who follow out genetic heritage and perform a large volume of exercise [1]. Despite the development of significant diagnostic and therapeutic medical options, the incidence of both diseases rose steadily through the first 70 years of the 20th century and began to decline after the 1962 publication of the United States Surgeon General’s recommendations against smoking [2] and Cooper’s Aerobics in 1968 [3], which essentially launched the “jogging” movement. The evidence supporting the positive health benefit of regular exercise is based on an abundant data base of epidemiological studies [4,5,6,7,8]. More recent population studies have demonstrated a dose–response effect of exercise on the risk of mortality, with an apparent saturation of benefit at ~20 Metabolic Equivalent Task (MET) hours per week [9,10,11,12,13,14]. There is, paradoxically, a slight excess in mortality above ~75 MET hours per week [9,10,11]. This is paralleled by a progressive reduction in the probability of cardiovascular events with increases in the number of steps accumulated per day [12,13,14].

With any health/medical therapy, particularly with drugs and surgery, there are usually iatrogenic (negative) side effects that need to be accounted for, which influences the dose of exercise recommended. In the 19th century, exercise (particularly athletics) was often viewed with some suspicion [15,16,17]. A series of reports, based on data collected after the beginning of the “jogging revolution” following the 1968 publication of Cooper’s Aerobics, suggested that middle-aged joggers were at somewhat of an increased risk during exercise [17]. This risk was largely seen to be based on a changed flow–demand relationship in the coronary circulation and the risk of rupturing of existing atherosclerotic plaque. Negative outcomes during exercise training appear to be based on a variety of factors (Table 1) [18]. In adults, early studies suggested that bad outcomes during exercise were most often seen in persons with underling coronary artery disease [17], and that the presentation of myocardial infarction vs. sudden cardiac death seemed to be related to the presence/absence of prior myocardial infarction (e.g., prior myocardial scarring) [17]. In the 1990s, a series of studies focused on the “triggering” of myocardial infarction, noted that when exercise was involved, it was usually “unaccustomed heavy (>6 METs) exercise” in previously sedentary individuals” [17]. Across time, the risk of untoward events during exercise training, whether in healthy individuals or patients with known cardiovascular disease, has generally decreased [15], perhaps largely because we have become better at recognizing when not to begin an exercise program, and by recognizing the importance of controlling intensity during the early days/weeks of an exercise program [15].

## 2. Exercise Testing

Graded exercise testing is a fundamental technique within medical diagnostics, fitness assessment, performance diagnostics, and exercise prescription [18,19,20]. There are a variety of reasons for exercise testing (Table 2) [18]. Exercise testing, which at the minimum usually involves progressively harder exercise, with electrocardiogram (ECG) and hemodynamic monitoring, can be augmented in a variety of ways including measurement of respiratory gas exchange, and methods designed to measure myocardial perfusion or ventricular function [19]. Exercise capacity derived from graded exercise tests has been shown to be very useful in terms of defining prognosis [21,22]. In middle-aged individuals, including patients with known cardiovascular disease, peak exercise capacities of >8 METs are associated with 5-year survival of ~95%, which gives physicians the latitude to try less invasive therapies. From the standpoint of prescribing exercise, there is a long tradition of using either the relative heart rate or heart rate reserve, or a normalized approach to exercise capacity (e.g., % heart rate (HR) reserve (% HRR), or % maximal oxygen consumption (% VO_2_ max) or maximal METs) [18,19]. For example, in two patients with the same evidence of a disease process (e.g., ST segment depression on the ECG, correlated with chest pain) may be viewed, and treated, very differently depending on their prognosis estimated from exercise capacity. 

In most cases, maximal exercise is often employed to allow optimization of the diagnostic sensitivity of exercise testing [23] and anchoring of the exercise prescription. However, submaximal testing has been shown to be a valuable alternative when maximal testing is not possible [24] and is certainly less demanding for the patient, may be perceived as safer, and does not require physician involvement. Submaximal exercise outcomes, such as the ventilatory threshold (VT), have become recognized as effective criteria for sustainable exercise capacity [25,26]. Exercise testing in clinical populations is generally thought to be quite safe, with complications requiring medical intervention occurring in ~1% of tests [27] and in healthy individuals/athletes is nearly zero [28]. More recent approaches to using the Rating of Perceived Exertion (RPE) [24,29] and the Talk Test [24,30] to evaluate exercise capacity have appeared and may be just as effective for exercise prescription. For example, an RPE rating of 13-14 or the first time speech comfort is “equivocal” is very close to the VT [24,30]. To the degree that VT may be just as good of an index of sustainable exercise capacity as VO_2_ max, such submaximal exercise testing options hold great promise.

## 3. Exercise Advice vs. Prescription

The value of exercise as a health promoting behavior is large enough that professional societies such as the American College of Sports Medicine, the American Heart Association, and the Centers for Disease Control and Prevention have issued guidelines for public behavior [31,32]. These guidelines, which carry a very favorable benefit–risk ratio, recommend that all adults accumulate at least 150 min of moderate (almost always < VT) intensity exercise, preferably distributed over at least 5 days per week. If one includes ordinary activities as well, this would represent ~10,000 steps per day [12,13,14] or ~20 METs hours per week [9,10,11]. It is important to note that these levels of recommendation, which probably approximate 7 h per week, are in excess of the 150 min per week recommended by professional societies [31,32]. The difference may be based on the belief that compliance to recommendations of 150 min per week is likely to be higher than to >400 min per week, and that the public health benefit of more people doing less than idealized exercise is larger than better grounded recommendations which may have lower compliance. These recommendations are not very individually tailored. In many ways, they are comparable to the traditional health recommendations, “an apple a day keeps the doctor away”, but have a likely large benefit in terms of health risk, with very minimal likelihood of untoward complications.

However, many people prefer more individually driven exercise prescription. Or in individuals with more fragile clinical conditions, more individually specific advice might be of value. Consistent with the concept of Exercise is Medicine^®^ promoted by the American College of Sports Medicine, there would be some sort of exercise-based evaluation that would allow the generation of an individual exercise prescription. The concept of the American College of Sports Medicine is based on the FITT-VP concept (frequency, intensity, time, type, volume, and progression) [18]. The most difficult of these elements to prescribe is intensity. In the original concept, the intensity of exercise was prescribed based on % of maximal HR (% HRmax), % HRR, % METs, or % MET Reserve [18]. This practice was supported by an abundant data base from randomized trials. However, it requires the presence of a maximal exercise test to anchor the HRmax or max METs, particularly since age-based population estimates of HRmax are known to be individually inadequate [33]. As early as the late 1970s, there were also reports suggesting that exercise prescriptions built on the so-called “relative percent concept” were not very good at creating a homogenous training experience and response [34,35]. Beyond this, it is widely recognized that HRmax achieved during a single incremental bout of exercise (particularly during tests conducted for clinical diagnostics) is unlikely to represent a true HRmax achieved during field testing, or interval training. More recent recommendations have suggested that exercise prescription concepts based on ventilatory/lactate threshold might be superior [25,26,36]. However, determination of “threshold” is technologically demanding, and requires a maximal exercise test. Recent work from our laboratory has suggested that the Talk Test may offer a technologically simple approach to VT estimation [30]. In the Talk Test, it is common to have subjects recite a standard speech passage of ~90 words, and then respond to the question, “can you speak comfortably?”. When the subject responds with “yes”, they are typically below the intensity of the VT. The first equivocal response, “yes, but”, usually occurs at about the intensity of the VT. If the subject responds with a definite “no”, the intensity is typically close to the respiratory compensation threshold [30]. Further, work with the RPE has suggested a low-tech way to evaluating either VT or VO_2_ max and prescribing exercise that is useful for recommending the intensity of training [25,37,38].

## 4. Functional Translation

One of the complicating issues when prescribing exercise training, particularly based on responses during submaximal or maximal exercise testing, is that the HR or RPE or Talk Test response at a particular workload during the exercise test “drifts” as exercise is sustained during training. This may be attributable to progressive changes in core temperature, to accumulation of catecholamines, to progressive dehydration, or to other factors related “to fatigue”. In other words, a workload that elicits a HR of 130 during exercise testing may have a HR of 145 after 30 min, secondary to cardiovascular drift. This leads to the practical problem of advising patients what to do in the gymnasium based on their exercise test responses. Imagine a person with a resting HR of 50, a maximal HR of 150, and thus a target HR at 70% HRR of 120, with an RPE of 13 and a Talk Test response of “yes, I can talk comfortably”. Let us say that this person achieves this HR at 6 min into a standard Bruce treadmill protocol (2.5 mph (4.0 kmh), 12% grade). However, if they go to the gymnasium and begin exercise training at this workload, their HR will quickly be greater than 120, their RPE will be 15 or more, they will not be comfortable talking, and they will have to quit after 15 min or so. Clearly, a prescribed workload will “drift” beyond the scope intended. This highlights the importance of monitoring responses during exercise training via multiple mechanisms (% HRR, RPE, Talk Test). However, sometime the monitoring methods have enough lag that the beginning exerciser gets “behind the curve” and becomes overly fatigued. In an experienced exerciser, this is not a problem. However, in the more vulnerable exerciser, it could provide the substrate for (at least) an unpleasant exercise session, if not an untoward event. If the exercise test responses could be “translated” such that the target workload designed to achieve target values for HR, RPE and Talk Test could be adjusted (down regulated) before the session begins then the response during training might be more optimal. 

We have taken this approach with ambulation following a standard treadmill test [38], with cycling after a standard cycle test [38], during arm–leg ergometry [39], during recreational activities [40] for target HR. We have also taken the same approach using the Talk Test as the outcome measure in sedentary individuals [41], well-trained individuals [42], and cardiac patients [30,43,44]. However, the magnitude of the “step down” (down regulation) in workload from exercise testing to exercise training is highly individual and depends on the details of the exercise test protocol. For example, in more rapidly ramped protocols, or in standard clinical protocols (e.g., Bruce treadmill protocols), the lack of near steady state conditions during testing makes the necessity for down-regulating the training session more difficult.

**Figure 1:**
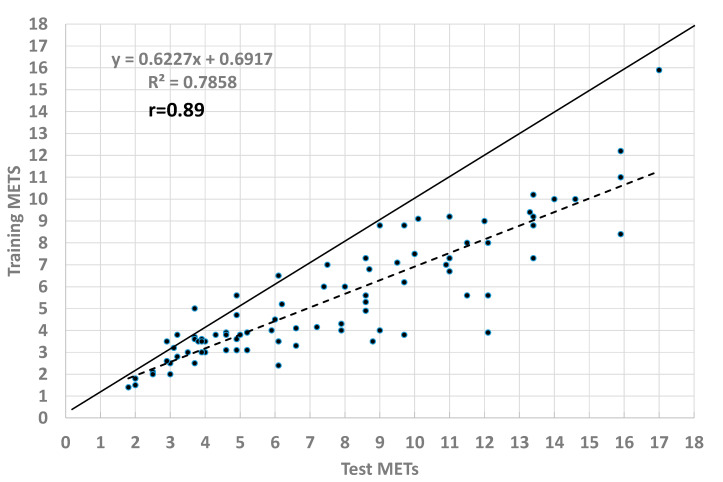
Comparison of exercise intensity (expressed as Metabolic Equivalent Task (METs) during exercise testing and exercise training with the same level of objective exercise intensity (% of Heart Rate Reserve, Rating of Perceived Exertion, Talk Test) based on combined results from several studies [38,39,40,41,42,43,44].

In this paper, we have taken the strategy of trying to “generalize” the magnitude of step down, by computing the relative workload, expressed as METs, on the basis of standard metabolic formulas [18] during both exercise testing and training, based on our previously published data [38,39,40,41,42,43,44]. We then plotted the METs during the exercise test against the METs during the steady state period (15–30 min) during exercise training, with the same magnitude of homeostatic disturbance in terms of % HRR, Talk Test score, and RPE. The results of this comparison are presented in Figure 1. The translated MET values were well correlated (r = 0.89) and the mean “step down” followed a linear regression, with an average step-down to 71.8% from testing (7.66 ± 4.03 METs) to training (5.50 ± 2.92 METs). This strategy, of course, is primarily designed for near steady state training sessions. In principle it could be extrapolated to interval training, although factors such as the length of the hard and easy segments, as well as the difference between hard and easy segments would have to be considered. Accordingly, the purpose of this study was to develop a ‘generalized model’ for translating exercise test responses to exercise prescription.

## 5. Discussion

The main finding of this viewpoint was that it appears possible to generalize previous studies [38,39,40,41,42,43,44] intended to “translate” exercise test responses into exercise prescriptions, by expressing the workload as METs. Using this approach, it appears that steady state exercise training at 65–75% of the workload yielding a particular marker of exercise intensity (i.e., % HRR, RPE, Talk Test) during exercise testing will yield comparable responses during exercise training. This problem has not otherwise been widely addressed. However, our results suggest that a larger “stepdown” than the ~10% recommended by Mezzani et al. [24] is necessary. The results are also consistent with the finding of de Koning et al. [45] that VT occurs at about 50% of peak power output (usually ~70% VO_2_ max) and that most exercise training, whether for athletes [26] or non-athletes [19], takes place at intensities <VT [20]. Validation of the magnitude of “stepdown” awaits further prospective data.

In the case of already active individuals, the spontaneous choice of the exercise level often yields intensities within commonly recommended guidelines [18]. Indeed, using the Talk Test as a surrogate of exercise intensity, athletic subjects required relatively little adjustment of workload beyond the first moments of the exercise bout [30,42,43]. In other words, in already experienced exercisers, finding the right intensity is relatively simple and rapid. Further, even in minimally trained individuals and cardiac patients, adjustment of the exercise level on the basis of speech comfort yielded appropriate relative MET and % HRR values within a very few minutes of exercise [30,44]. We also know that previously untrained individuals can self-regulate exercise intensity using the RPE scale and achieve an effective training response [37].

In sedentary individuals, unaccustomed heavy exercise can be associated with the “triggering” of myocardial infarction [17]. However, we also know that the incidence of exercise related complications, both in healthy individuals and in cardiac rehabilitation programs, has decreased over time [15], most likely because we are better at regulating exercise intensity during the early weeks of new exercise programs (e.g., avoiding conditions that might promote “triggering” of myocardial infarction). Against the background that many people who have been sedentary for many years and who are beginning an exercise program later in life, and that they may tend to exercise at intensities recalled from earlier in their life. This workload may now be too strenuous and may predispose toward the development of untoward events during training. Thus, rather than prescribing exercise on the basis of % HRR or even RPE, which may take some time to show that the exercise is too strenuous, it would seem to be desirable to have a mechanism for deciding what the appropriate workload should be during the first few days of training, and then fine tuning (e.g., triangulate) this intensity on the basis of conventional monitoring tools (% HRR, RPE, Talk Test).

## 6. Conclusions

The present results suggest the utility of “translating” exercise test responses into the workload during exercise training that will yield appropriate levels of exercise intensity as defined by % HRR, RPE, or Talk Test. It appears that reducing the workload to 65–75% of that during the exercise test is reasonable and can be done on the basis of METs calculated using standard methods [18]. Particularly in sedentary individuals beginning an exercise program or in patients during rehabilitation, this approach may yield useful estimates of exercise intensity and contribute to both the safety and efficacy of exercise therapy. These data suggest a simple approach to using exercise testing results, which are meaningfully generalized [38,39,40,41,42,43,44] from our earlier more specific results. Hopefully, this makes the “functional translation” approach easier to use, although this new approach requires future experimental verification.

## Figures and Tables

**Table 1:** Negative outcomes during exercise training.

Congenital Abnormalities
Hypertrophic cardiomyopathy
Arrhythmogenic RV dysplasia
Coronary artery anomalies
**Undiagnosed Coronary Artery Disease**
Pre-exercise screening identifies >50%
First presentation of cardiovascular disease is often fatal
33% males, 12% females
**Drug Use**
Anabolic steroids
Stimulants
Erythropoietin
Recreational drugs
**Trauma**
Commotio cordis

**Table 2:** Reasons for exercise testing.

Evaluate Exertional Discomfort
Reduced exercise tolerance
Chest pain
Dyspnea
Claudication
Cerebral symptoms
**Reveal Occult Pathology**
Change presentation of cardiovascular disease
**Define Prognosis**
Guide to exercise prescription strategy
**Exercise Prescription**
Relative percentage concept
Ischemic/arrhythmic threshold
